# Effect of the structure and volume fraction of nanophase on the mechanical properties of (FeNi)_86_B_14_ amorphous nanocrystalline alloys

**DOI:** 10.1038/s41598-024-56442-2

**Published:** 2024-07-08

**Authors:** Yiran Zhang, Jing Pang, Qingchun Xiang, Dong Yang, Yinglei Ren, Xiaoyu Li, Keqiang Qiu

**Affiliations:** 1 School of Materials Science and Engineering, Shenyang University of Technology, Shenyang, 110870 China; 2 Qingdao Yunlu Advanced Material Technology Co. Ltd., Qingdao, 266232 China

**Keywords:** Nanoscale materials, Theory and computation

## Abstract

The influence of precipitated nanophases on the mechanical properties of Fe-based amorphous nanocrystalline alloys is an urgent issue to be explored. Two amorphous nanocrystalline alloys, i.e., (Fe_0.9_Ni_0.1_)_86_B_14_ and (Fe_0.7_Ni_0.3_)_86_B_14_ containing nanophase of the body-centered cubic and face-centered cubic structures, respectively, were selected to investigate the effect of the structure and volume fraction of nanophase on their mechanical properties. The results of nanoindentation experiments and the calculation of the volume and size of the shear transition zone reveal that the two alloys show different mechanical properties. When the volume fraction of the nanophase in (Fe_0.9_Ni_0.1_)_86_B_14_ is larger than 50%, the elastic modulus is increased suddenly and the volume and size of the shear transition zone is decreased dramatically, while no dramatic change occurs in (Fe_0.7_Ni_0.3_)_86_B_14_. Moreover, it was found by using molecular dynamics simulations that the main reason for these abnormal mechanical properties is the change of cluster type in the system due to the incorporation of nanophases with different structures.

## Introduction

In recent years, Fe-based amorphous nanocrystalline alloys have been widely used due to their excellent soft magnetic properties^1–4^. Annealing and quenching processes are common techniques to control the microstructure and properties of engineering materials^5^. This method is very effective in producing amorphous nanocrystalline soft magnetic alloys, i.e., Fe-based amorphous nanocrystalline soft magnetic materials are first prepared by producing amorphous ribbons as precursors by fast-cooling the liquid metal and then by precipitating nanocrystalline grains within the amorphous ribbons by using suitable annealing heat treatments^6,7^. Furthermore, it is also found that the nonferromagnetic elements in the amorphous precursors can accelerate the nucleation frequency and inhibit the crystal growth process during nanocrystallization^8^. It can be seen that amorphous nanocrystalline alloys consist of two parts, i.e., amorphous matrix and nanocrystalline phases. In addition, the structure and volume fraction of nanophases can be controlled by changing the composition and the annealing process. Therefore, the structure and the volume fraction of nanophase affect the mechanical properties of amorphous nanocrystalline alloys.

According to previous studies^9–12^, alloys with a single face-centered cubic (FCC) structure usually have high plasticity but low yield strength, however, alloys with a single body-centered cubic (BCC) structure have high strength but low plasticity. Meanwhile, it has been shown that the nanoscale FCC-Al particles in an Al-based amorphous matrix cause a drastic increase in tensile fracture strength to 1560 MPa, which is three times higher than that of conventional high-strength-type Al-based alloys. The reason for this result is the increase in the number of nanoscale icosahedral clusters due to the incorporation of the FCC-Al phase, resulting in mechanical properties superior to those of commercial high-strength Al-based crystal alloys^13^. Moreover, due to the extreme difficulty of experimental observation and the complexity of the microstructure of amorphous nanocrystalline alloys, it is common to resort to molecular dynamics (MD) simulations to probe this structure at the atomic level and the changes in the clusters^14–18^. Therefore, it can be seen that the mechanical properties of alloys are influenced by the microstructure. Do the different structure and volume fractions of nanophases ultimately impact the overall mechanical properties of Fe-based amorphous nanocrystalline alloys? Presently, little investigation has been conducted on this issue. For the currently mature Fe-based amorphous nanocrystalline alloys that are capable of precipitating different structural nanocrystalline compositions by annealing, the Fe–Ni–B system^19^ is possible to answer this question. Moreover, for the Fe–Ni–B amorphous nanocrystalline alloy system, the transformation of ferromagnetic BCC-(Fe, Ni) nanocrystals to FCC-(Fe, Ni) nanocrystalline phases may occur by increasing Ni content. Meanwhile, according to Kajiwara et al.^20^, it was found that the residual austenitic FCC-(Fe, Ni) nanocrystals could not be easily transformed into martensitic BCC-(Fe, Ni) phase when further cooled from room temperature to 77 K. Therefore, quenching in liquid nitrogen was chosen in this work to ensure the stable presence of the FCC-(Fe, Ni) phase.

In this work, (Fe_1-*x*_Ni_*x*_)_86_B_14_ (*x* = 0.1, 0.3) was selected as the material to explore the changes in structure and volume fractions of nanophases for amorphous nanocrystalline alloys by using quenching and annealing heat treatments. Moreover, the mechanical properties of the two alloys were examined by nanoindentation experiments. Meanwhile, the effect of nanocrystal incorporation on the clusters in the system was revealed by using MD simulation.

## Results

**Figure 1:**
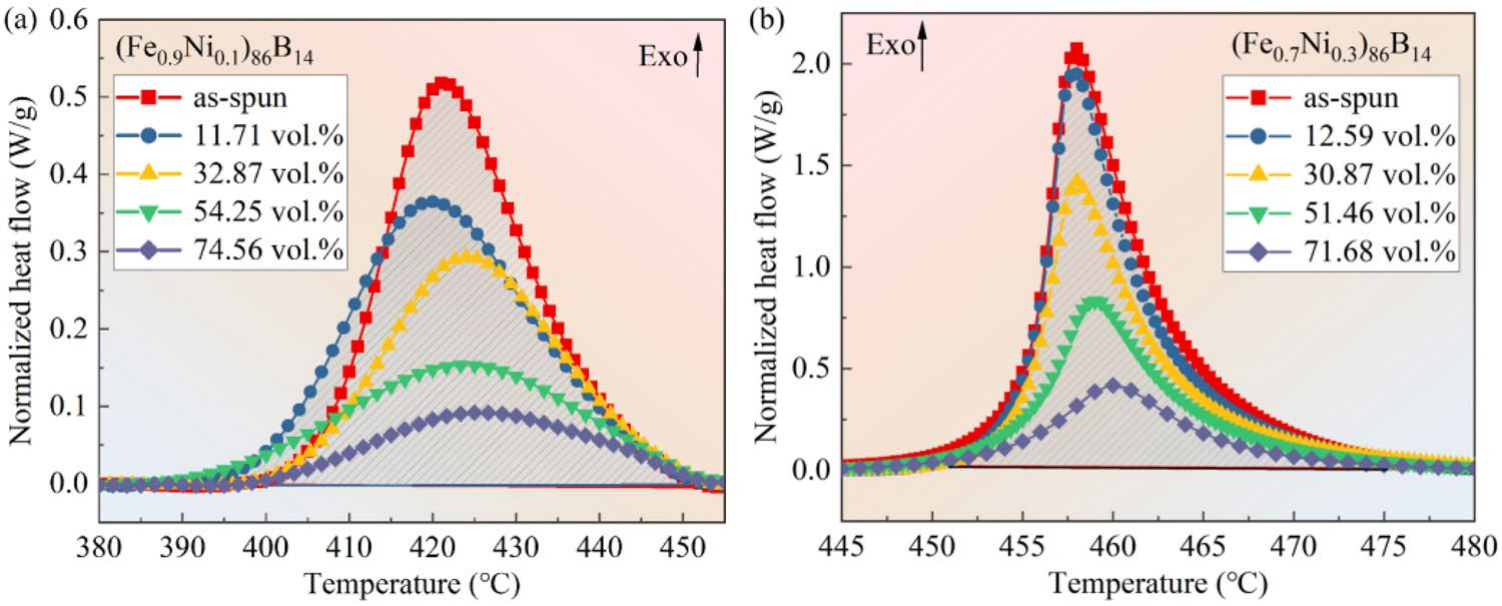
DSC curves of (**a**) (Fe_0.9_Ni_0.1_)_86_B_14_ and (**b**) (Fe_0.7_Ni_0.3_)_86_B_14_ samples, respectively.

In order to obtain the thermodynamic parameters of the samples, differential scanning calorimetry (DSC) tests were carried out on the as-spun and as-annealed ribbon type samples as shown in Fig. 1. Where only the exothermic peak corresponding to Fe nanophase with different volume fractions is shown for different samples. The volume fractions of Fe nanophase obtained by crystallization (*V*_*cry*_) of the amorphous samples can be calculated from the enthalpy of crystallization using Eq. (1) as follows^6^:

1 $${V}_{cry}=\frac{\Delta {H}_{ca}-\Delta {H}_{cn}}{\Delta {H}_{ca}}\times 100\mathrm{\%}$$

where Δ*H*_*ca*_ is the enthalpy of the crystallization of the Fe nanophases for the as-spun sample, and Δ*H*_*cn*_ is the enthalpy of crystallization of the Fe nanophases for the annealed treatment sample. As shown in Fig. 1, the upward arrow is the exothermic direction, and the volume fraction Fe nanophases for each annealed state sample was obtained by using Eq. (1). Two amorphous nanocrystalline alloys, i.e., (Fe_0.9_Ni_0.1_)_86_B_14_ and (Fe_0.7_Ni_0.3_)_86_B_14_, with similar *V*_*cry*_, were obtained by controlling the annealing time. For (Fe_0.9_Ni_0.1_)_86_B_14_, the *V*_*cry*_ of Fe nanophase is 11.71, 32.87, 54.25, and 74.56%, respectively, as shown in Fig. 1a. While the *V*_*cry*_ of Fe nanophase is 12.59, 30.87, 51.46, and 71.68%, respectively, for (Fe_0.7_Ni_0.3_)_86_B_14_ annealed ribbon. It can be seen that the crystallization peak of Fe phases decreases with the increase the volume fraction, indicating that the more Fe crystalline phases precipitate in the amorphous matrix.

**Figure 2:**
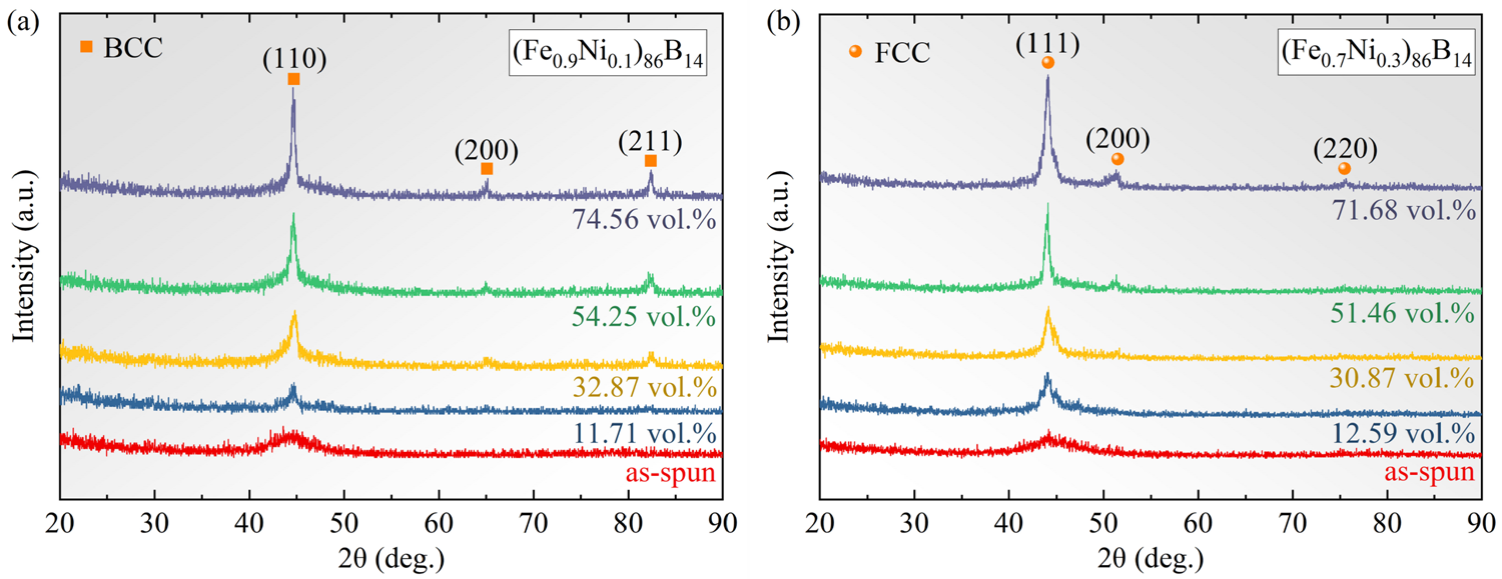
XRD curves of (**a**) (Fe_0.9_Ni_0.1_)_86_B_14_ and (**b**) (Fe_0.7_Ni_0.3_)_86_B_14_ samples, respectively.

Figure 2 shows the X-ray diffraction (XRD) curves of the samples in the as-spun and as-annealed states. It is indicated that the two as-spun alloy ribbons are in the fully amorphous state. According to Fig. 2a, the patterns of the annealed (Fe_0.9_Ni_0.1_)_86_B_14_ samples show distinct diffraction peaks at 2θ≈45, 65, and 83°, which correspond to the (110), (200), and (211) characteristic planes of the BCC-(Fe, Ni) or α-Fe (Ni) phase, respectively. While the patterns of the annealed (Fe_0.7_Ni_0.3_)_86_B_14_ samples show obvious diffraction peaks at 2θ≈44, 51, and 75° as shown in Fig. 2b, which correspond to the (111), (200), and (220) characteristic planes of the FCC-(Fe, Ni) or γ-Fe (Ni) phase. The different locations of the apparent diffraction peaks indicate that the two types of crystallization phases with different structures were precipitated after the rapid annealing treatment.

**Figure 3:**
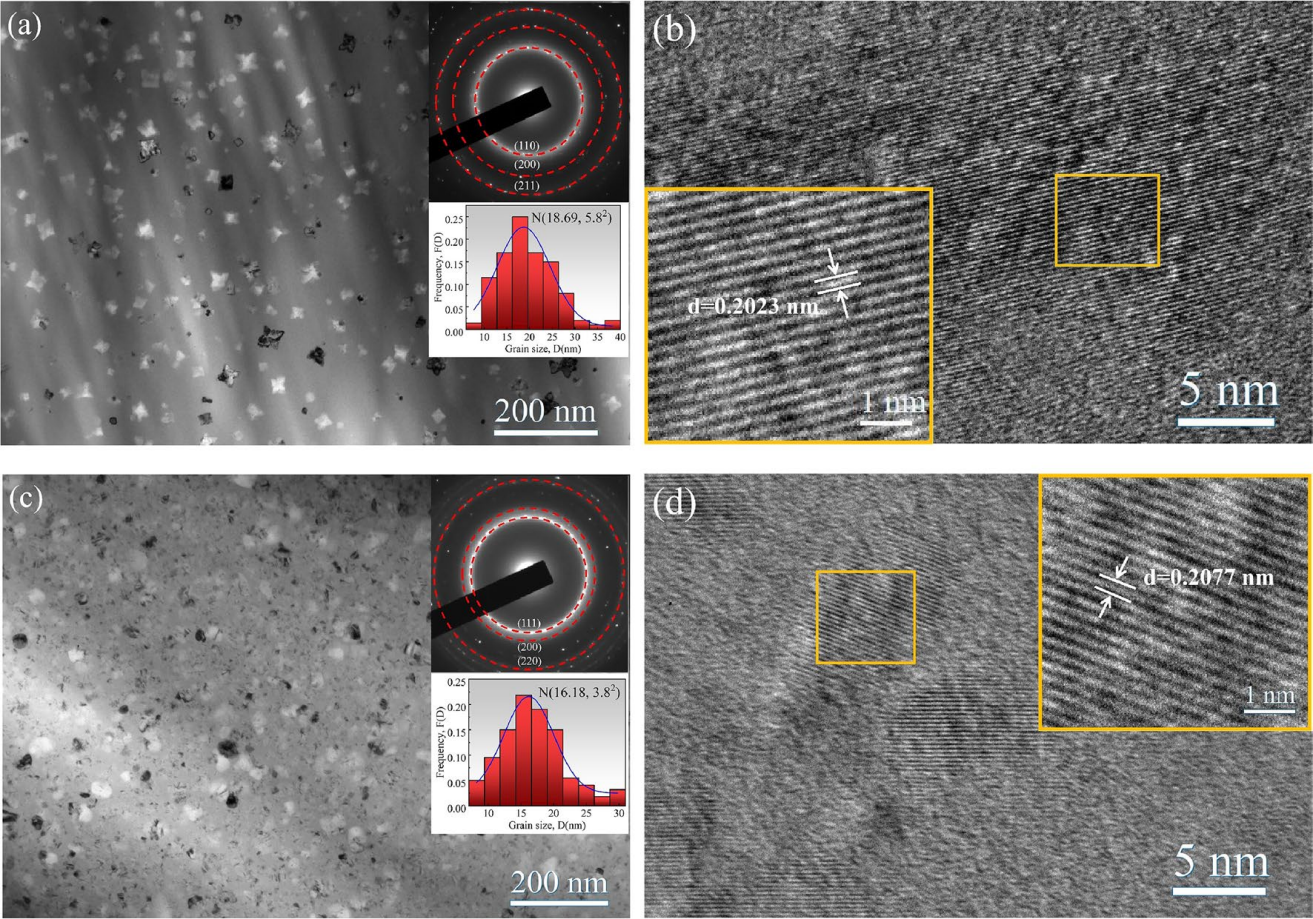
TEM images for (**a**) (Fe_0.9_Ni_0.1_)_86_B_14_ with a volume fraction of 54.25%, and (**c**) (Fe_0.7_Ni_0.3_)_86_B_14_ with a volume fraction of 51.46%, respectively. The insets in (**a**) and (**c**) show the corresponding SAED patterns (up-right corner), as well as grain size distribution and standard deviation with Gaussian fitting (down-right side). The partially enlarged views for (**a**) and (**c**) are indicated by (**b**) and (**d**), respectively.

The changes in thermal response and the structure of the as-annealed ribbons can be seen in the curves of DSC and XRD, but the size and distribution of the nanocrystals in the amorphous matrix were not visualized, so the samples were examined by transmission electron microscopy (TEM). The TEM image of (Fe_0.9_Ni_0.1_)_86_B_14_ amorphous nanocrystalline alloy with a volume fraction of 54.25% is shown in Fig. 3a, and the insets represent the histograms of the selected area electron diffraction (SAED) spectra (in the up-right) and the histograms of grain size distribution (in the down-right), respectively. It can be seen that the statistically averaged nanocrystalline size is 18.69 nm, the interplanar spacing is obtained by the SAED calculations, and the corresponding crystal faces are the (110), (200), and (211) from inside to outside, respectively, which are the same as the results obtained in Fig. 2a. The enlarged area of Fig. 3a is shown in Fig. 3b, from which we can see that the interplanar spacing of (110) is 0.2023 nm, further indicating that the nanophase is BCC-(Fe, Ni). Figure 3c shows the TEM image of (Fe_0.7_Ni_0.3_)_86_B_14_ amorphous nanocrystalline alloy with a volume fraction of 51.46%. The average grain size is 16.18 nm, and the crystallographic facet indices are (111), (200) and (220) obtained by SAED from inside to outside. Meanwhile, as shown in Fig. 3d, the enlarged area of Fig. 3c, the interplanar spacing of (111) is 0.2077 nm, which is in agreement with the results obtained from the XRD patterns shown in Fig. 2b, i.e., the FCC-(Fe, Ni) nanophase is precipitated in the matrix of (Fe_0.7_Ni_0.3_)_86_B_14_ amorphous alloy after annealing.

**Figure 4:**
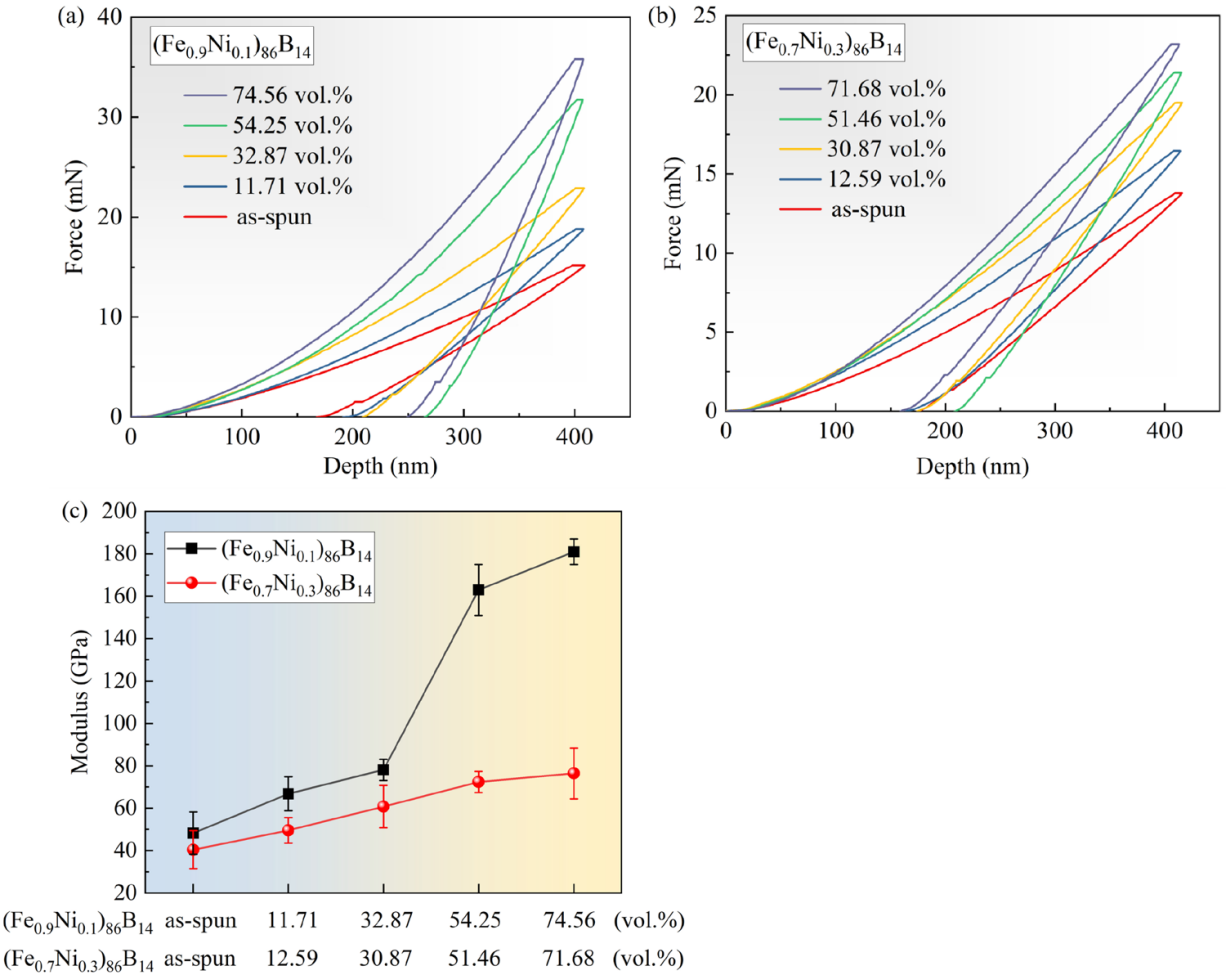
Force-depth curves for (**a**) (Fe_0.9_Ni_0.1_)_86_B_14_, and (**b**) (Fe_0.7_Ni_0.3_)_86_B_14_ samples, respectively, and (**c**) the curves of elasticity modulus vs volume fractions of nanophase for the alloy samples.

In order to further investigate the mechanical properties of the two amorphous nanocrystalline alloys, nanoindentation tests were carried out, as shown in Fig. 4. The curves of force-depth for (Fe_0.9_Ni_0.1_)_86_B_14_ are shown in Fig. 4a, and it can be seen that there is a steep increase in force when the volume fraction α-Fe (Ni) nanophase is larger than 54.25%. In contrast, the force loaded into (Fe_0.7_Ni_0.3_)_86_B_14_ amorphous nanocrystalline alloy, shown in Fig. 4b, shows a gradual increase in force with the increase in volume fraction of γ-Fe (Ni) nanophase without steep increase. Meanwhile, the changes of elastic modulus with volume fraction of nanophases for the two alloys are plotted in Fig. 4c, which clearly shows that there is a steep increase in the elastic modulus of (Fe_0.9_Ni_0.1_)_86_B_14_ when the volume fraction of α-Fe (Ni) nanophase is larger than 50%, which leads to an increase in brittleness of the alloy because an increase in the elastic modulus indicates an enhancement in stiffness^18^. For (Fe_0.7_Ni_0.3_)_86_B_14_ amorphous nanocrystalline alloy, however, the increase in volume fraction of γ-Fe (Ni) nanophases does not have a significant effect on the brittleness. This result indicates that the amorphous nanocrystalline alloy containing FCC-(Fe, Ni) nanophase, even if its content is larger than 50 vol.%, has better toughness compared to the amorphous nanocrystalline alloy precipitating BCC-(Fe, Ni) nanophase.

**Figure 5:**
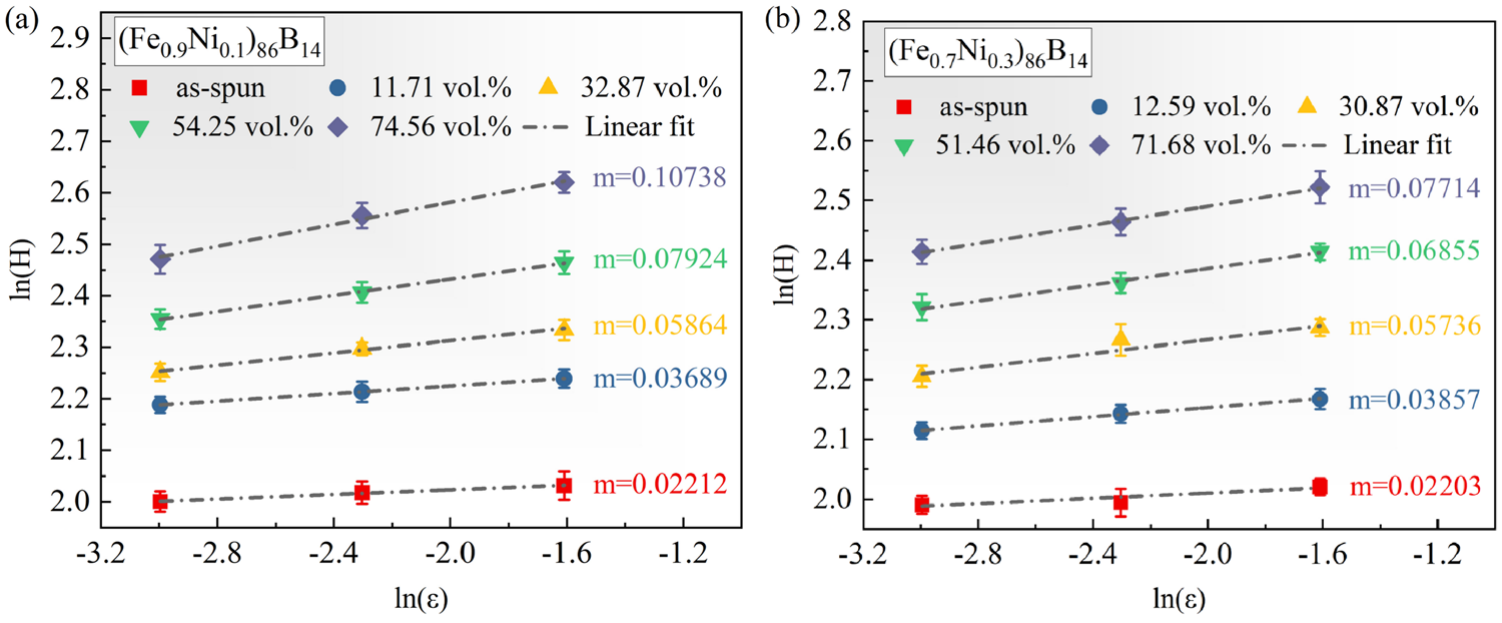
The strain rate sensitivity of hardness for (**a**) (Fe_0.9_Ni_0.1_)_86_B_14_ and (**b**) (Fe_0.7_Ni_0.3_)_86_B_14_ samples, respectively.

In order to explore the effect of volume fraction on the toughness of the two amorphous nanocrystalline alloys more intuitively, according to the shear transition zone (STZ) model^21–24^, the STZ volume (Ω) can be calculated with the following equation^22^:

2 $$\Omega =\frac{\mathrm{\kappa T}}{\mathrm{C^{\prime}}{\text{mH}}}$$

where κ is the Boltzmann constant, T is room temperature, H is the hardness by indention, m is the index of the strain rate sensitivity, and C′ is calculated using the following equation^22–24^:

3 $$\frac{{\uptau }_{{\text{CT}}}}{{\uptau }_{{\text{C}}}}=1-\frac{0.016}{0.036}{\left(\frac{{\text{T}}}{{{\text{T}}}_{{\text{g}}}}\right)}^{0.62}$$

4 $$\mathrm{C{\prime}}=\frac{2\mathrm{R\xi }}{\sqrt{3}}\frac{{{\text{G}}}_{0}{\upgamma }_{{\text{C}}}^{2}}{{\uptau }_{{\text{C}}}}{\left(1-\frac{{\uptau }_{{\text{CT}}}}{{\uptau }_{{\text{C}}}}\right)}^\frac{1}{2}$$

where T_g_ is the glass transition temperature, and R, ξ, γc, and $\frac{{\uptau }_{{\text{C}}}}{{{\text{G}}}_{0}}$ are constants of 0.25, 3, 0.027, and 0.036, respectively. Meanwhile, m values can be obtained from the slope between the hardness and the strain rate, as shown in Fig. 5. As can be seen that the m values of (Fe_0.9_Ni_0.1_)_86_B_14_ and (Fe_0.7_Ni_0.3_)_86_B_14_ samples are similar for volume fractions of Fe nanophase less than 50%. When the value is larger than 50 vol. %, the m values of (Fe_0.9_Ni_0.1_)_86_B_14_ show a sudden increase, which is larger than the corresponding ones of (Fe_0.7_Ni_0.3_)_86_B_14_. In addition, to obtain the STZ size, i.e., the number of atoms contained in the STZ, it is necessary to calculate the STZ size R, which can be obtained by Eq. (5)^23^:

5 $${\text{R}}={\left({\sum }_{{\text{i}}}^{{\text{n}}}{{\text{A}}}_{{\text{i}}}{{\text{r}}}_{{\text{i}}}^{3}\right)}^\frac{1}{3}$$

where A_i_ and r_i_ are the atomic fraction and atomic radius of each element, respectively. The m, Ω, and STZ size of (Fe_0.9_Ni_0.1_)_86_B_14_ and (Fe_0.7_Ni_0.3_)_86_B_14_ samples, subsequently, were calculated using the aforementioned information and the results are presented in Table 1. As can be seen in Table 1, at the volume fraction of nanophase being of 0%, the values of Ω for (Fe_0.9_Ni_0.1_)_86_B_14_ and (Fe_0.7_Ni_0.3_)_86_B_14_ samples are 3.574 nm^3^ and 3.6544 nm^3^, corresponding to STZ sizes of 497 and 508, respectively. Furthermore, when the volume fraction of nanophase increases to approximately 30% for the two alloys, the STZ volume decreases to 1.0246 nm^3^ and 1.0910 nm^3^, while the STZ size decreases to 142 and 151, respectively. Notably, there is little distinction between the values of the two alloys. The STZ volume and size decrease as the volume fractions of nanophases increase due to the formation and concentration of STZ being closely correlated with the free volume. Therefore, the decrease in the STZ volume and size indicates the reduction in the free volume in the alloy. However, it is noteworthy that when the volume fraction of nanophase is increased to larger than 50 vol.%, significant differences in the STZ volume and size of (Fe_0.9_Ni_0.1_)_86_B_14_ and (Fe_0.7_Ni_0.3_)_86_B_14_ amorphous nanocrystalline alloys are observed. Relative to the STZ size values of 113 and 91 for (Fe_0.7_Ni_0.3_)_86_B_14_, the values for (Fe_0.9_Ni_0.1_)_86_B_14_ decrease significantly to 94 and 60, which suggests that the formation of nanocrystals with BCC structure is more inhibitory to the free volume for volume fractions of Fe (Ni) nanophases larger than 50%.

**Table 1:** Summary of STZ data for (Fe_0.9_Ni_0.1_)_86_B_14_ and (Fe_0.7_Ni_0.3_)_86_B_14_ samples.

Alloy	Volume fractions	m	*Ω* (nm^3^)	STZ size (atom)
(Fe_0.9_Ni_0.1_)_86_B_14_	0 vol.%	0.02212	3.574	497
	11.71 vol.%	0.03689	1.7653	245
	32.87 vol.%	0.05864	1.0246	142
	54.25 vol.%	0.07924	0.6759	94
	74.56 vol.%	0.10738	0.4329	60
(Fe_0.7_Ni_0.3_)_86_B_14_	0 vol.%	0.02203	3.6544	508
	12.59 vol.%	0.03857	1.8149	252
	30.87 vol.%	0.05736	1.0910	151
	51.46 vol.%	0.06855	0.8155	113
	71.68 vol.%	0.07714	0.6548	91

## Discussion

**Figure 6:**
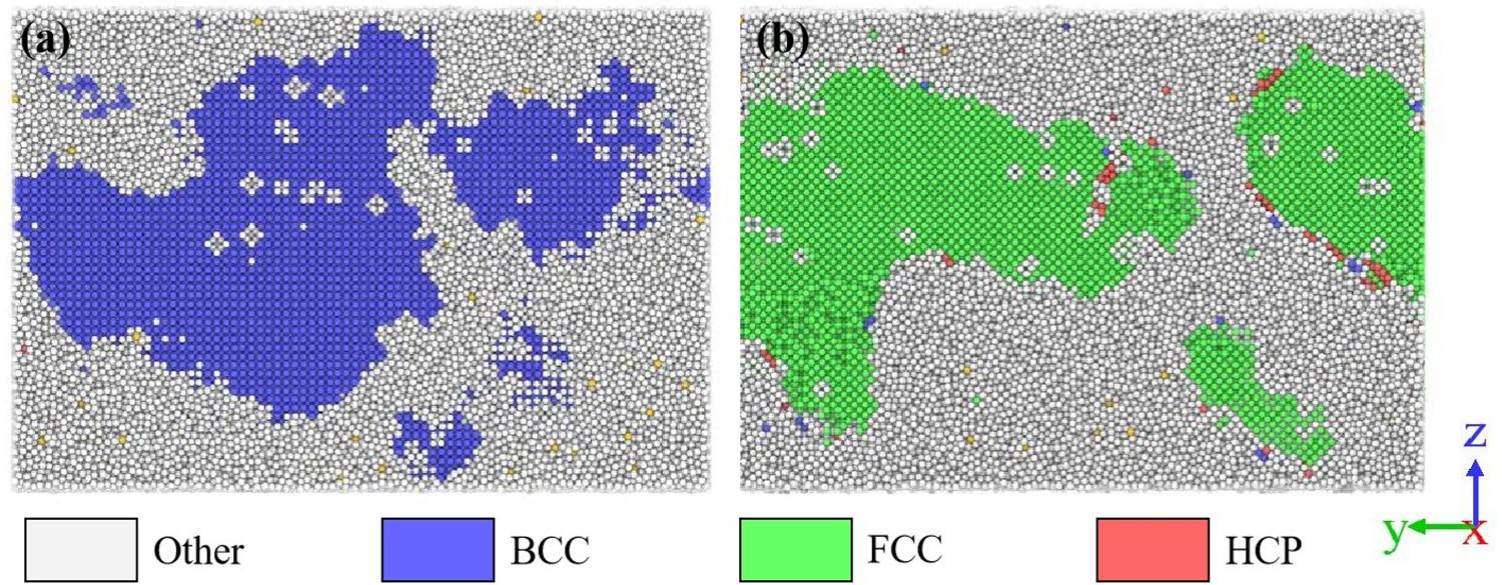
The model cross-section of (**a**) Fe_90_Ni_10_ and (**b**) Fe_70_Ni_30_, respectively.

To investigate the effect of the structure of nanophases on the overall properties of amorphous nanocrystalline alloys when the volume fractions of nanophases are larger than 50%, MD simulations have resorted to exploring the changes of the free volume in amorphous nanocrystalline alloys. Fe_90_Ni_10_ and Fe_70_Ni_30_ amorphous nanocrystalline alloys were constructed using large-scale atomic/molecular massive parallel simulator (LAMMPS) and the atomic configurations were visualized by common neighbor analysis (CNA) in open visualization tool (OVITO) software^25^. As shown in Fig. 6, the white color atoms represent the amorphous structure, the blue color atoms are the BCC structure, the green color atoms are the FCC structure, and the red color atoms are the HCP structure. Meanwhile, Fig. 6a shows the cross-section of Fe_90_Ni_10_ model, where the embedded crystals are the BCC structure and the whole system contains the BCC crystals with a volume fraction of 54.6%. However, as shown in Fig. 6b for the cross-section of Fe_70_Ni_30_ model, the whole system contains crystals of FCC structure with a volume fraction of 52.0%.

**Figure 7:**
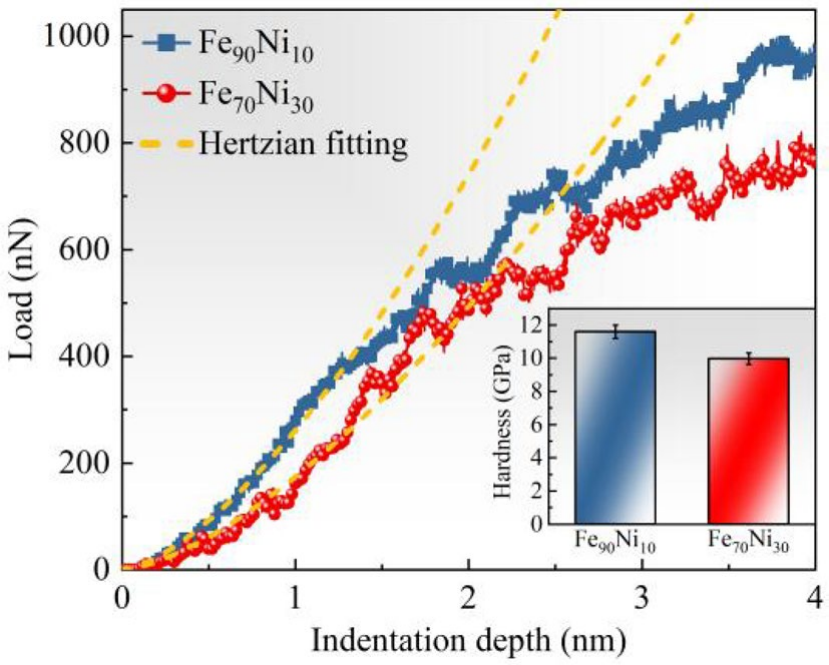
Load-depth curves of the nanoindentation for the Fe_90_Ni_10_ and Fe_70_Ni_30_ model, and the inset showing the average hardness of the indentation depth of 2.5–5 nm.

The above models are simulated for the nanoindentation process, and the load-depth (P–h) curves can be obtained, as shown in Fig. 7. It can be seen that the load of Fe_90_Ni_10_ is larger than that of Fe_70_Ni_30_ when the indentation depth is 4 nm, which is the same as the results obtained from experiments (Fig. 4a and b). Furthermore, at the nanoscale, the elastic range of the nanoindentation curve can be well fitted by the Hertzian behavior^26^, which can be expressed as:

6 $${\text{P}}=\frac{4}{3}{{\text{E}}}_{*}{{\text{R}}}^{1/2}{{\text{h}}}^{3/2}$$

where P is the load, R is the radius of the indenter, h is the depth of the indentation, and E_*_ is the simplified Young's modulus. Since a virtual rigid indenter is used in the current MD simulation, the elastic modulus of the sample can be calculated:

7 $${{\text{E}}}_{{\text{s}}}=(1-{{\text{v}}}_{{\text{s}}}^{2}){{\text{E}}}_{*}$$

where v_s_ and E_s_ are the Poisson's ratio and the Young's modulus of the samples, respectively. Using Eqs. (6) and (7), the values of E_s_ for the two models Fe_90_Ni_10_ and Fe_70_Ni_30_ are calculated to be 170.81 and 76.02 GPa, respectively, with the same trend as the results obtained in Fig. 4c. Meanwhile, the indentation hardness is calculated to better observe the mechanical behavior in the case of different structures on the overall mechanical behavior of amorphous nanocrystalline alloys. The formulation of Meyer hardness (H) is expressed as follows^27^:

8 $${\text{H}}=\frac{{\text{P}}}{\uppi (2{\text{R}}-{\text{h}}){\text{h}}}$$

As shown in the inset of Fig. 7, the hardness calculated using Eq. (8) indicates the hardness of the Fe_70_Ni_30_ model is less than that of the Fe_90_Ni_10_ model, which maintains the consistency with the results obtained in Fig. 5.

**Figure 8:**
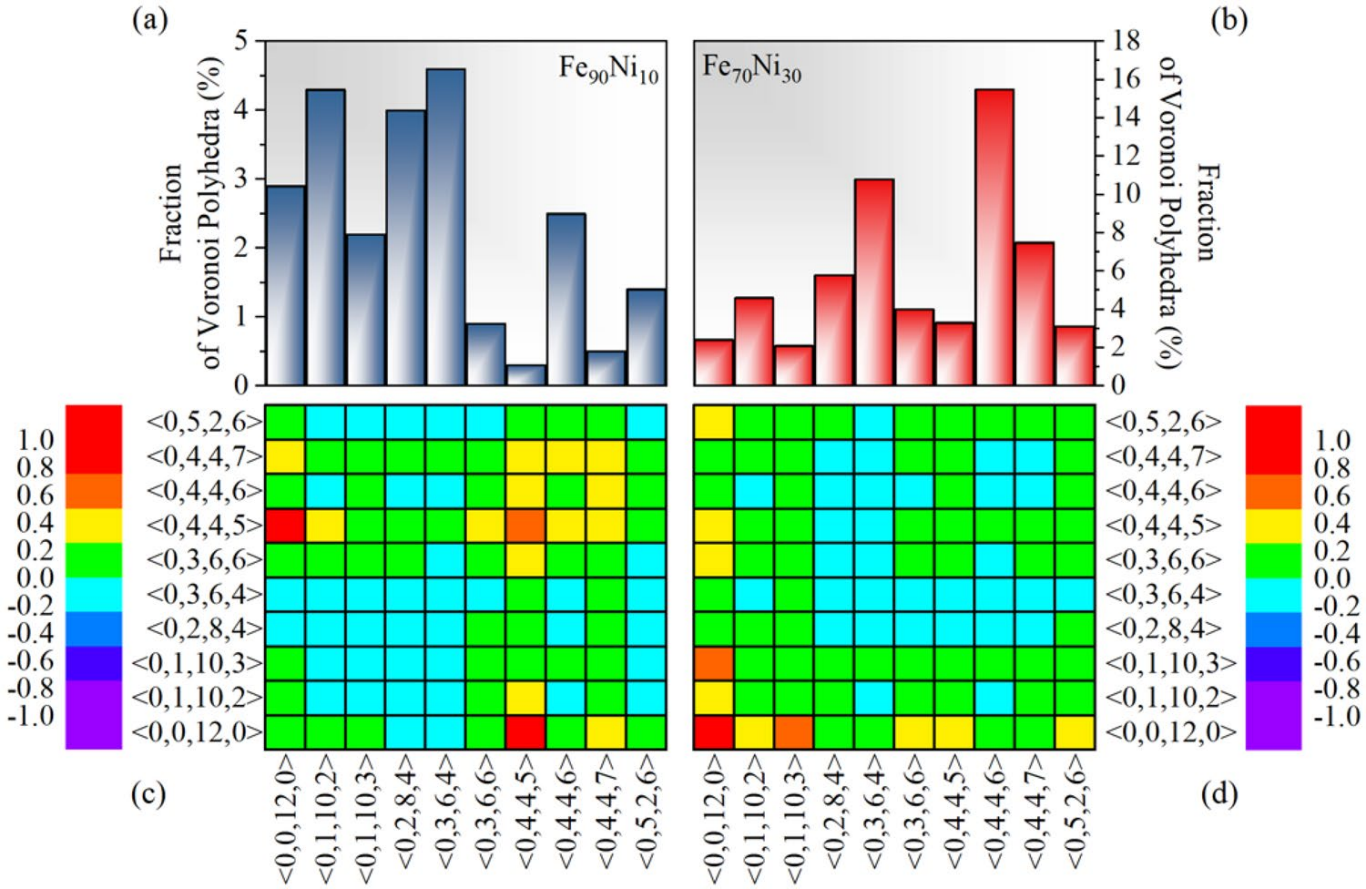
The percentages of Voronoi polyhedra in Fe_90_Ni_10_ (**a**) and Fe_70_Ni_30_ (**b**), and the matrix of spatial correlation index *C*_*ij*_ for the Voronoi polyhedra in Fe_90_Ni_10_ (**c**) and Fe_70_Ni_30_ (**d**) amorphous nanocrystalline alloys.

The results obtained from the simulation have the same trend as the experimental results, indicating the reasonableness of the modeling. The difference in macroscopic mechanical properties is caused by the difference in the structure of the spatial arrangement of atoms. At present, the atomic Voronoi polyhedral structure analysis algorithm is widely used to reflect the free volume in the sample, whose index and scale content directly affect the mechanical properties of the material^16,28–30^. The occupancy percentages of the individual Voronoi indices of the two models are shown in Fig. 8a and b, which shows that the alloy models containing different crystal structures have different Voronoi indices occupying the highest percentage. As shown in Fig. 8a, the Fe_90_Ni_10_ model containing the BCC crystal structure occupies the highest percentage of Voronoi indices are  < 0,3,6,4 > , and this index is typical of BCC-like structures. However, the relative comparison reveals that the Fe_70_Ni_30_ model containing the FCC crystal structure occupies the highest percentage of Voronoi indices of  < 0,4,4,6 > , which is related to FCC symmetry, as shown in Fig. 8b. It can be seen that the reason for the significant difference for the percentage of the Voronoi index in the system is due to the different nanocrystalline structures. Meanwhile, in order to investigate the spatial correlation of the different types of Voronoi polyhedra of the two models, the nearest neighbor correlation index *C*_*ij*_ between the central atoms of the two Voronoi polyhedra of types *i* and *j* can be calculated by using the following formula^15^:

9 $${C}_{ij}=\frac{{P}_{ij}}{{P}_{ij}^{0}}-1$$

where *P*_*ij*_ is the probability of Voronoi polyhedra types *i* and *j* being the nearest neighbors, and ${P}_{ij}^{0}$ is the probability of the randomized spatial distribution for polyhedra types *i* and *j*. The equations for *P*_*ij*_ and ${P}_{ij}^{0}$ are as follows:

10 $${P}_{ij}=\frac{{m}_{ij}}{{P}_{total}}$$

11 $${P}_{ij}^{0}=\left\{\begin{array}{c}\frac{2{n}_{i}{n}_{j}}{N\left(N-1\right)} \left(i\ne j\right)\\ \frac{{n}_{i}\left({n}_{i}-1\right)}{N\left(N-1\right)} \left(i=j\right)\end{array}\right.$$

where *m*_*ij*_ is the number of the nearest neighbors of types *i* and *j*, *P*_*total*_ is the total number of the nearest neighbor pairs in whole model, *n*_*i*_ (*n*_*j*_) is the number of atoms of type *i* (*j*), and *N* is the total number of atoms in the whole model. By this definition, substituting Eqs. (10) and (11) into Eq. (9), if *C*_*ij*_ is zero, it indicates that polyhedra types *i* and *j* tend to be randomly space distributed. Meanwhile, the larger positive value of *C*_*ij*_ indicates that the central atoms of polyhedra types *i* and *j* are more likely to form nearest neighbor atom pairs, i.e., the correlation between the two Voronoi indices is stronger. Conversely, *C*_*ij*_ is less than zero, which indicates that the central atoms of polyhedra types *i* and *j* tend to repel each other and avoid becoming nearest neighbor atoms. As shown in Fig. 8c and d, the correlation matrix of *C*_*ij*_ for the two models is calculated by using Eq. (9) in this work, where the color from red to purple indicates the correlation from strong to weak in order. It can be seen that these two models containing different nanocrystalline structures have different Voronoi indices with high correlation strength. As shown in Fig. 8c, there is a strong correlation between  < 0,0,12,0 >  and  < 0,4,4,5 > , however, in Fig. 8d, the index that has a strong correlation with  < 0,0,12,0 >  besides itself is the  < 0,1,10,2 >  Voronoi index. Furthermore, according to the Refs^18,31^, it is known that based on topological differences, individual Voronoi polyhedra can be categorized into three groups, crystal-like, icosahedral-like, and intermediate. Meanwhile,  < 0,4,4,5 >  belongs to the crystal-like index, which has more four- and six-translational symmetries, however, both  < 0,0,12,0 >  and  < 0,1,10,2 >  belong to the icosahedral-like indices, which mainly have more five-translational symmetries. It is well known that the topology of amorphous alloys is mainly composed of  < 0,0,12,0 >  full icosahedron and  < 0,1,10,2 >  can also be considered as icosahedral-like since it can be formed by  < 0,0,12,0 > . It can be seen that when the volume fractions of nanophases with different structures are larger than 50% the changes in the percentage and correlation of the Voronoi index in the system occur due to incorporation of different types of Voronoi polyhedra, and this is the reason for the significant differences in the macroscopic mechanical properties of amorphous nanocrystalline alloys.

## Conclusions

For (FeNi)_86_B_14_ amorphous nanocrystalline alloys containing nanophases with different structures, when the volume fraction of nanophases is less than 50%, (Fe_0.9_Ni_0.1_)_86_B_14_ containing BCC-(Fe, Ni) nanophase and (Fe_0.7_Ni_0.3_)_86_B_14_ containing FCC-(Fe, Ni) nanophase have similar elastic modulus. However, a sudden increase in the elastic modulus of (Fe_0.9_Ni_0.1_)_86_B_14_ amorphous nanocrystalline alloy occurs when the nanophase is larger than 50 vol.%. It is found that the volume and size of STZ decrease with the increase of the volume fraction of nanophase. And those values of STZ become drastically decreased for (Fe_0.9_Ni_0.1_)_86_B_14_, but not for (Fe_0.7_Ni_0.3_)_86_B_14_, when the volume fraction of nanophase is larger than 50%. MD simulations reveals that the structure of nanocrystals in the two models causes a change in the cluster type. The  < 0,3,6,4 >  Voronoi index of BCC-like structure plays a dominant role in (Fe_0.9_Ni_0.1_)_86_B_14_, whereas in (Fe_0.7_Ni_0.3_)_86_B_14_, it is the Voronoi index of the FCC-like structure  < 0,4,4,6 >  that plays a dominant role, and this is what causes the two amorphous structures to change, which is the main reason for the different macroscopic properties of the two amorphous nanocrystalline alloys. It can be seen that when the volume fraction of nanocrystals is larger than 50%, the mechanical properties of the material become poor and cannot be restored by rejuvenation methods. In such cases, the study of materials mainly considers functionality, such as soft magnetic properties, rather than mechanical properties. However, when the volume fraction of the nanocrystals is less than 50%, the mechanical properties of the alloy are dominated by the amorphous matrix, and the poor mechanical properties of the as-annealed material can be recovered by the rejuvenation methods, i.e., cryogenic thermal cycling treatment is beneficial to improve the toughness of amorphous alloys. Therefore, the volume fraction threshold provided in this paper is an important guide for the subsequent study and application of amorphous nanocrystalline alloys.

## Methods

### Experimental procedures

Alloy ingots were produced with nominal compositions of (Fe_1-*x*_Ni_*x*_)_86_B_14_ (*x* = 0.1, 0.3) through arc melting of a mixture of Fe (99.8 wt. %), Ni (99.9 wt. %), and pre-alloyed Fe-B (19.35 wt. % B) materials in a high-purity argon atmosphere. The resulting ingots were then broken into pieces and remelted in quartz tubes with nozzles through inductive melting. Subsequently, alloy ribbons, with a width of approximately 2 mm and a thickness of around 20 μm, were prepared through the single-roller melt spinning method at a tangent speed of 48 m/s. The resulting crystallization process and thermal stability of the alloy ribbons were examined through DSC (TA-Q100) at a heating rate of 20 K/min under a nitrogen atmosphere, from which the glass transition temperature (*T*_*g*_), and the crystallization temperatures (*T*_*x*_) were determined. The annealing temperatures of (Fe_0.9_Ni_0.1_)_86_B_14_ and (Fe_0.7_Ni_0.3_)_86_B_14_ alloys were selected to be 693 K and 730 K, respectively, and the annealing times ranged from 60 to 300 s. The annealing process involved sandwiching amorphous ribbons between a pair of copper sheets before placing them in a preheated Zn-Sn metal melt and then rapidly cooling them in liquid nitrogen. After different annealing time, amorphous nanocrystalline alloys with different structures and volume fractions of nanophases were obtained. The phase constituents of both as-spun and as-annealed ribbons were determined using XRD (Shimadzu, 7000S) with Cu-Kα radiation (λ = 1.54056 Å, E = 8.049 keV). Additionally, the microstructure of the as-annealed ribbons was analyzed using TEM (JEM-2100F). The TEM sample was prepared by ion milling (Gatan 691). First, the thinning was carried out at a large angle of 8°/min, and then when a small hole appeared in the center of the sample, it was immediately switched to a small angle of 4°/min for repairing the hole to obtain as much thin area as possible. Nanoindentation tests (KLA-Tencor, Nano Indenter G200) were performed using a Berkovich diamond indenter in static displacement mode, with a spacing of 50 µm between indentations, a maximum displacement of 400 nm, and loading and unloading strain rates of 0.02 s^−1^, 0.05 s^−1^, and 0.1 s^−1^, respectively, and retention time of 15 s.

### Molecular dynamics simulations

The nanoindentation simulations were performed by MD simulations and all MD simulations were based on an open-source program, namely LAMMPS^32^. In the case of metals, interatomic interactions are typically described using the embedded atomic method (EAM) potential^33–35^. The EAM potential developed by Bonny et al.^36^ in the Fe–Ni binary system was used in present simulation, which has been proven to be highly effective^10,37,38^ in other Fe–Ni dominated systems. Over 700,000 atoms were simulated for the Fe_90_Ni_10_ and Fe_70_Ni_30_ systems, respectively, with dimensions of 24 × 24 × 17 nm^3^. The simulation involved heating the system from 300 to 3000 K with periodic boundary conditions and allowing it to relax at 3000 K for 300 ps to ensure sufficient melting. Subsequently, rapid cooling to 800 K at 10^12^ K/s was performed, and then crystals were randomly embedded throughout the model to simulate the process of nanoprecipitation, with BCC structured nanocrystals in Fe_90_Ni_10_ and FCC structured nanocrystals in Fe_70_Ni_30_, respectively. The system was then relaxed for 100 ps before being quenched to 300 K to minimize potential energy between the crystal and amorphous interfaces. The entire simulation was carried out with isobaric-isothermal (NPT) conditions and at zero pressure. Furthermore, the above model was used to simulate the nanoindentation process under the microcanonical ensemble (NVE) system. For all simulations, all results were visualized using OVITO^39^.

## Data Availability

All data generated or analyzed in this study are included in this published article or are available from the corresponding author upon reasonable request.

## References

[1] Cao CC (2018). Local structure, nucleation sites and crystallization behavior and their efects on magnetic properties of Fe_81_Si_*x*_B_10_P_8-__*x*_Cu_1_ (*x*=0-8). Sci. Rep..

[2] Kim W, Duan T, Perepezko JH (2023). Nanocrystal evolution during ultra-fast heating in an amorphous Fe_85_B_15_ alloy. Scripta Mater..

[3] Liu T (2021). Heterostructured crystallization mechanism and its effect on enlarging the processing window of Fe-based nanocrystalline alloys. J. Mater. Sci. Technol..

[4] Silveyra JM, Ferrara E, Huber DL, Monson TC (2018). Soft magnetic materials for a sustainable and electrified world. Science.

[5] Pradeep KG, Herzer G, Choi P, Raabe D (2014). Atom probe tomography study of ultrahigh nanocrystallization rates in FeSiNbBCu soft magnetic amorphous alloys on rapid annealing. Acta Mater..

[6] Meng Y, Pang SJ, Chang CT, Bai XY, Zhang T (2021). Nanocrystalline Fe_83_Si_4_B_10_P_2_Cu_1_ ribbons with improved soft magnetic properties and bendability prepared via rapid annealing of the amorphous precursor. J. Magn. Magn. Mater..

[7] Sun YY, Song M, Liao XZ, Sha G, He YH (2012). Effects of isothermal annealing on the microstructures and mechanical properties of a FeCuSiBAl amorphous alloy. Mat. Sci. Eng. A.

[8] Li Z (2020). Dramatic grain refinement and magnetic softening induced by Ni addition in Fe B based nanocrystalline soft magnetic alloys. Scripta Mater..

[9] Yavari AR (2002). FeNiB-based metallic glasses with FCC crystallisation products. J. Non-Cryst. Solids.

[10] Byshkin M, Hou M (2012). Phase transformations and segregation in Fe-Ni alloys and nanoalloys. J. Mater. Sci..

[11] Qin W, Chen ZH (2001). Stability of the austenitic phase in ultra-fine particles of Fe-based alloys. J. Alloy. Compd..

[12] Varillas J, Ocenášeka J, Torner J, Alcalá J (2021). Understanding imprint formation, plastic instabilities and hardness evolutions in FCC, BCC and HCP metal surfaces. Acta Mater..

[13] Inoue A, Kimura H (2001). Fabrications and mechanical properties of bulk amorphous, nanocrystalline, nanoquasicrystalline alloys in aluminum-based system. J. Light Metals.

[14] Liu XJ (2008). Atomistic mechanism for nanocrystallization of metallic glasses. Acta Mater..

[15] Li MZ, Wang CZ, Hao SG, Kramer MJ, Ho KM (2009). Structural heterogeneity and medium-range order in Zr_*x*_Cu_100-__*x*_ metallic glasses. Phys. Rev. B.

[16] Yu Q, Wang XD, Lou HB, Cao QP, Jiang JZ (2016). Atomic packing in Fe-based metallic glasses. Acta Mater..

[17] Lu LL (2023). Co-evolution of the atomic/nano scale structural heterogeneities and magnetic properties of Fe-Si-B-Cu metallic glass during relaxation with composition fluctuations. J. Non-Cryst. Solids.

[18] Cheng YQ, Ma E (2011). Atomic-level structure and structure-property relationship in metallic glasses. Prog. Mater. Sci..

[19] Li Z (2020). Dramatic grain refinement and magnetic softening induced by Ni addition in Fe-B based nanocrystalline soft magnetic alloys. Scripta Mater..

[20] Kajiwara S, Ohno S, Honma K (1991). Martensitic transformations in ultra-fine particles of metals and alloys. Philos. Mag. A.

[21] Ma Y, Peng GJ, Debela TT, Zhang TH (2015). Nanoindentation study on the characteristic of shear transformation zone volume in metallic glassy films. Scripta Mater..

[22] Johnson WL, Samwer K (2005). A universal criterion for plastic yielding of metallic glasses with a (T/T_g_)^2/3^ temperature dependence. Phys. Rev. Lett..

[23] Pan D, Inoue A, Sakurai T, Chen MW (2008). Experimental characterization of shear transformation zones for plastic flow of bulk metallic glasses. Proc. Natl. Acad. Sci. USA.

[24] Zheng H, Zhu L, Jiang SS, Wang YG, Chen FG (2019). Recovering the bending ductility of the stress-relieved Fe-based amorphous alloy ribbons by cryogenic thermal cycling. J. Alloy. Compd..

[25] Tsuzuki H, Branicio PS, Rino JP (2007). Structural characterization of deformed crystals by analysis of common atomic neighborhood. Comput. Phys. Commun..

[26] Qiu C, Zhu PZ, Fang FZ, Yuan DD, Shen XC (2014). Study of nanoindentation behavior of amorphous alloy using molecular dynamics. Appl. Surf. Sci..

[27] Zhao HW (2012). Research on the effects of machining-induced subsurface damages on mono-crystalline silicon via molecular dynamics simulation. Appl. Surf. Sci..

[28] Zhou SX (2012). The relationship between the stability of glass-forming Fe-based liquid alloys and the metalloid-centered clusters. J. Appl. Phys..

[29] Jiang Q (2020). Atomic-level understanding of crystallization in the selective laser melting of Fe_50_Ni_50_ amorphous alloy. Addit. Manuf..

[30] Peng HL, Li MZ, Wang WH (2011). Structural signature of plastic deformation in metallic glasses. Phys. Rev. Lett..

[31] Sheng HW, Luo WK, Alamgir FM, Bai JM, Ma E (2006). Atomic packing and short-to-medium-range order in metallic glasses. Nature.

[32] Plimpton SJ (1995). Fast parallel algorithms for short-range molecular dynamics. J. Comput. Phys..

[33] Daw MS, Baskes MI, Daw MS, Baskes MI (1983). Semiempirical, quantum mechanical calculation of hydrogen embrittlement in metals. Phys. Rev. Lett..

[34] Cheng YQ, Cao AJ, Ma E (2009). Correlation between the elastic modulus and the intrinsic plastic behavior of metallic glasses: The roles of atomic configuration and alloy composition. Acta Mater..

[35] Shi Y, Falk ML (2007). Stress-induced structural transformation and shear banding during simulated nanoindentation of a metallic glass. Acta Mater..

[36] Bonny G, Pasianot RC, Malerba L (2009). Fe-Ni many-body potential for metallurgical applications. Model. Simul. Mater. Sci..

[37] You LJ, Hu LJ, Xie YP, Zhao SJ (2016). Influence of Cu precipitation on tensile properties of Fe-Cu-Ni ternary alloy at different temperatures by molecular dynamics simulation. Comp. Mater. Sci..

[38] Cui W, Peng C, Wang L, Qi Y, Fang T (2014). Structure and dynamics of undercooled FeNi. Phys. Chem. Liq..

[39] Stukowski A (2010). Visualization and analysis of atomistic simulation data with OVITO-the open visualization tool. Model. Simul. Materi. Sci..

